# Multi-atlas multi-modality morphometry analysis of the South Texas Alzheimer’s Disease Research Center postmortem repository

**DOI:** 10.1016/j.nicl.2025.103752

**Published:** 2025-02-18

**Authors:** Nicolas Honnorat, Mariam Mojtabai, Karl Li, Jinqi Li, David Michael Martinez, Tanweer Rashid, Morgan Smith, Margaret E Flanagan, Elyas Fadaee, Morgan Fox Torres, Mallory Keating, Kevin Bieniek, Sudha Seshadri, Mohamad Habes

**Affiliations:** a Neuroimage Analytics Laboratory and Biggs Institute Neuroimaging Core, Glenn Biggs Institute for Neurodegenerative Disorders, University of Texas Health Science Center at San Antonio, San Antonio, TX, USA; b Department of Pathology, University of Texas Health Science Center at San Antonio, San Antonio, TX, USA; c Research Imaging Institute, University of Texas Health Science Center at San Antonio, San Antonio, TX, USA

**Keywords:** Postmortem MRI, Registration, Deep learning

## Abstract

Histopathology provides critical insights into the neurological processes inducing neurodegenerative diseases and their impact on the brain, but brain banks combining histology and neuroimaging data are difficult to create. As part of an ongoing global effort to establish new brain banks providing both high-quality neuroimaging scans and detailed histopathology examinations, the South Texas Alzheimer’s Disease Re- search Center postmortem repository was recently created with the specific purpose of studying comorbid dementias. As the repository is reaching a milestone of two hundred brain donations and a hundred curated MRI sessions are ready for processing, robust statistical analyses can now be conducted. In this work, we report the very first morphometry analysis conducted with this new data set. We describe the processing pipelines that were specifically developed to exploit the available MRI sequences, and we explain how we addressed several postmortem neuroimaging challenges, such as the separation of brain tissues from fixative fluids, the need for updated brain atlases, and the tissue contrast changes induced by brain fixation. In general, our results establish that a combination of structural MRI sequences can provide enough informa- tion for state-of-the-art Deep Learning algorithms to almost perfectly separate brain tissues from a formalin buffered solution. Regional brain volumes are challenging to measure in postmortem scans, but robust estimates sensitive to sex differences and age trends, reflecting clinical diagnosis, neuropathology findings, and the shrinkage induced by tissue fixation can be obtained. We hope that the new processing methods developed in this work, such as the lightweight Deep Networks we used to identify the formalin signal in multimodal MRI scans and the MRI synthesis tools we used to fix our anisotropic resolution brain scans, will inspire other research teams working with postmortem MRI scans.

## Highlights

• We conduct the first morphometry study of the South Texas ADRC postmortem repository.
• We compare neuroimaging findings with the neuropathology examinations available for this cohort.
• Our analysis demonstrates the robustness of our new image processing pipelines.
• We successfully detect the atrophy induced by healthy aging and dementia.
• We hope that our new Deep Learning tools and pipelines will be useful in the future.

## 1 Introduction

Histopathology was of critical importance to describe neurodegenerative disorders and understand how they affect the brain (Khachaturian, 1985, Hyman, 1997). The observation and the staining of postmortem tissue samples led to the description and the staging of Alzheimer’s Disease progression (Braak and Braak, 1991, Mirra et al., 1991). Neuropathology is also used to study and retrospectively assert a diagnosis of Lewy body Dementia (Mackenzie, 2000, McKeith et al., 2017) and was of key importance to characterize the Limbic-predominant age-related TDP-43 encephalopathy (LATE) (Nelson et al., 2019); to mention just a few salient examples.

Brain histopathology is still of great importance today (DeTure and Dickson, 2019, Shepherd et al., 2019). Unfortunately, brain samples are difficult to collect. To address this issue, universities and research agencies have recently started to join their efforts to create larger, sometimes collaborative brain tissue banks (Bell et al., 2008, Shepherd et al., 2019) and they are actively supporting the creation of new local and national repositories (Freund et al., 2018).

The very recent creation of the South Texas Alzheimer’s Disease Research Center repository is part of this global effort to keep producing neuroimaging and neuropathology data of increasing quality to open the way for new discoveries (Li et al., 2023). Unlike previous repositories that were often focusing on non-neurological controls (Jonkman et al., 2019) or small cohorts corresponding to a single pathology (Freund et al., 2018); and often missing postmortem neuroimaging data (Grinberg et al., 2007); the South Texas Alzheimer’s Disease Research Center repository was deliberately focused on the study of comorbid dementias via the acquisition of rich multimodal neuroimaging data and detailed neuropathology examinations. This design was adopted to account for the recent neuropathology findings suggesting a broad prevalence of comorbid neurodegenerative diseases (Knopman et al., 2003, Jellinger and Attems, 2010).

As the South Texas Alzheimer’s Disease Research Center repository is reaching two hundred brain donations (Li et al., 2023); a set of neuroimaging data sufficiently large for conducting robust statistical tests is now available for research and particularly for carrying out morphometry studies to measure and compare the volumes of various brain tissues and brain regions. We report in this work the findings of a multi-atlas brain morphometry study conducted for a first batch of 92 whole brain hemispheres. In vivo, antemortem MRI scans were available for 31 brain donors. They were parcellated to study the differences between in vivo and postmortem regional brain volumes. In addition, we exploited 42 white matter intensity masks manually delineated by our neurologists to characterize the distribution of these tissue alterations in the brain samples and improve the brain tissue maps automatically segmented by our pipelines. During our experiments, we identified a set of serious challenges raised by postmortem neuroimaging data, such as the need for ad-hoc postmortem brain segmentation algorithms and the limited spatial resolution of the MRI scans available in medical records. We explored strategies to overcome these issues, such as the use of deep networks combining T1-weighted and T2-weighted MRI signals to separate postmortem brain tissues from their surrounding formalin fixative, the use of brain atlases manually edited to better fit postmortem cerebellar regions, and the use of white matter hyperintensity masks to improve brain tissue segmentations. Overall, our results establish that a combination of structural MRI sequences can provide enough information for standard Deep Learning algorithms to almost perfectly separate brain tissues from a formalin buffered solution. Regional brain volumes were especially challenging to measure, but our estimations were robust and sensitive enough to capture sex differences, age trends, and the shrinkage induced by tissue fixation. We hope that the new processing methods developed in this work will be adopted to analyze additional postmortem neuroimaging repositories in the future.

## 2 Materials and methods

### 2.1 Data acquisition

The South Texas Alzheimer’s Disease Research Center postmortem repository was established by col- lecting brain samples donated at the University of Texas Health Science Center at San Antonio to study the effects of neurodegenerative disorders (Li et al., 2023). The brain bank focuses then on donors with a clinical diagnosis of dementia at death.

The collected brains, preserved in a 10 % neutral buffered formalin solution, are separated into two parts: the right hemisphere is frozen and preserved for future studies, while the left hemisphere is packed in a plastic bag filled with the formalin buffer, used to acquire a series of MRI scans including structural T1- weighted and T2-weighted MRI scans, and finally dissected to carry out a histopathology examination of various brain regions (Li et al., 2023). These histopathology examinations, covering 23 brain regions and conducted according to the NIA Alzheimer’s Association guidelines (Montine et al., 2012); aim at quantifying various biological factors to neurodegeneration and, in particular, at evaluating three neuropathology scores: the Thal phase reflecting the amount of amyloid-beta plaque deposition in the brain (Thal and U. Ru¨b, M. Orantes, H. Braak, 2002); the Braak stage evaluating the spread of neurofibrillary tangles in the brain (Braak and Braak, 1991), and the Consortium to Establish a Registry for Alzheimer’s Disease (CERAD) neuritic plaque score (Mirra et al., 1991). The reader is referred to (Li et al., 2023) for more details regarding the brain donation consent forms and the staining chemicals employed during the histopathology exams.

In addition and in preparation for this work, the neurologists working at the South Texas Alzheimer’s Disease Research Center examined the T2-weighted postmortem MRI scans to delineate white matter hyper- intensities, and the families were contacted to obtain brain MRI scans acquired while the brain donors were still alive. These efforts provided white matter hyperintensities masks for nearly half of the brain donors and in vivo scans for a third of the brain donors. The acquisition and the preparation of these neuroimaging and neuropathology data are detailed in the next Sections.

### 2.2 Postmortem data

All the scans were acquired using the Siemens TimTrio 3 T scanner belonging to the Research Imaging Institute of the University of Texas Health Science Center at San Antonio. As shown in our previous work (Li et al., 2023); the brains’ left hemispheres were packed in plastic bags filled with formalin and wrapped in absorbent pads and placed in a Siemens 8-channel knee coil during the scans. The size of this knee coil was perfect to hold the bags in place during the scans, and significantly increased the image quality (Li et al., 2023). The T1-weighted postmortem scans were acquired at a repetition time of 2200 ms, echo time 3.25 ms, flip angle 13*^◦^*, a spatial resolution of 0.5 x 0.5 x 0.5 mm, and averaged 4 times. The T2-weighted postmortem scans considered in this work were acquired at a repetition time of 3750 ms, echo time 50 ms, flip angle 120*^◦^*, a spatial resolution of 1.5 x 0.6 x 0.6 mm, and averaged 4 times.

After visual quality control, the postmortem MRI sessions of a total of 92 brain donors were retained for the present study. Three donors, two women and a man, had their postmortem brain scanned twice. A single pair of MRI scans was available for all the other donors. Table 1 summarizes the demographics of these 92 donors and indicates that there was no significant age difference between the groups of men and women donors (p-value 0.5 for Welch two-sample *t*-test), who were almost perfectly balanced in the sample (47 women for 45 men).

**Table 1:** Cohort of brain donors considered in this work. Neuropathology scores were missing for one woman (neuropath. subgroup), but available for the 42 donors with white matter hyperintensities masks (WMH subgroup) and the 31 donors with in vivo MRI scans (in vivo subgroup). The fixation time, during which the samples remained in formalin before being scanned, could be calculated for 84 brains.

subgroup	all	men	women	p
**all donors**	92	47	45	
age at death	77.08	77.81	76.32	0.50∗
**diagnosis**	51	28	23	0.73+
AD	7	4	3	1.00+
FTD	21	11	10	1.00+
OD	10	6	4	0.74+
missing	15	3	12	0.03+
**neuropathology**	91	47	44	1.00+
age at death	76.95	77.81	76.03	0.42∗
height (m)	1.69	1.76	1.61	**< 10** *^−^* ^15*^
sum of scores	10.35	10.86	9.8	0.23∗
Thal phase	3.81	4.06	3.55	0.14∗
Braak stage	4.62	4.78	4.45	0.38∗
CERAD	1.91	2.02	1.8	0.35∗
**WMH**	42	22	20	1.00+
age at death	76.72	76.86	76.57	0.92∗
lesions (*cm*^3^)	6.78	10.01	3.22	**0.003**∗
**in vivo**	31	19	12	0.41+
age at death	74.27	74.83	73.38	0.71∗
in vivo age	70.44	71.03	69.51	0.70∗
age gap	3.83	3.79	3.88	0.94∗
**fixation time**	84	46	38	0.65+
mean (days)	50.29	50.48	50.05	0.91∗

∗ Welch Two Sample *t*-test (men versus women).

+ Fisher exact test (subgroup versus all donors).

A clinical diagnosis could be established for 77 of these brain donors (15 missing diagnoses). The present study will focus on the four most frequent neurodegenerative diseases reported in that group: Alzheimer’s disease (AD, affecting 51 brain donors), vascular disease (VD, 10 donors), frontotemporal dementia (FTD, 7 donors), and other unspecified dementia (OD, 21 donors). 67 donors presented a single disease at the time of their death (41 men and 26 women), while 8 were suffering from two diseases (one man and seven women), and two men from three diseases. Due to the recruitment strategy of the South Texas Alzheimer’s Disease Research Center repository, all the donors were suffering from at least one dementia. Table 1 indicates that there was no imbalance between the diagnoses received by men and women, but the diagnosis was more often missing for women (12/15).

The fixation times, during which the brain samples remained in formalin before being scanned, could be calculated for 84 of our brain samples. These samples correspond to 46 men and 38 women, and this proportion of men and women is not statistically significantly different from the overall proportion of men and women in our study (p-value 0.65 for Fisher’s exact test). On average, the brain remained in formalin for fifty days (shortest fixation time: 11 days, longest fixation time 95 days, 90 % interval: 25 to 82 days), and the fixation time was almost similar for men and women according to the results reported at the bottom of Table 1. The reader is referred to our previous work introducing the South Texas Alzheimer’s Disease Research Center postmortem brain repository for more details (Li et al., 2023).

### 2.3 Neuropathology scores

Neuropathology scores could be obtained for all but one brain donor in our study sample (a woman). The CERAD scores were encoded into integer values as follows: a CERAD score corresponding to an absence of neuritic plaques was encoded as 0, sparse plaques were encoded as 1, a moderate presence of neuritic plaques with 2, and frequent plaques were encoded with the value 3. Table 1 reports no significant difference between the Thal, Braak, and CERAD scores measured for men and women. As a result, the sum of the three scores was also similar for men and women. The height of these brain donors was recorded as well, and the statistical analysis indicates that the women were significantly shorter than the men by fifteen centimeters on average (p-value 5.1 × 10^-16^).

### 2.4 White matter hyperintensities

Due to the large amount of time required to manually segment high-resolution MRI scans, white matter hyperintensities could only be delineated within the T2-weighted postmortem MRI scans of 42 brain donors, 22 men and 20 women. This proportion of men and women is almost the same as the overall gender distribution in the set of brain donors (p-value close to 1.0 when conducting a Fisher exact test), and the age at death of the brain donors with white matter hyperintensities masks was not significantly different between men and women (p-value 0.92, two sample T-test). A two-sample T-test indicated that white matter hyperintensities were significantly larger in men’s brains, with 10.01 cc on average compared to an average of 3.22 cc for women (p-value 0.0031). This p-value remains significant at level 0.05 after a correction for multiple comparisons carried out for the fifteen statistical tests reported in Table 1.

### 2.5 In vivo data

Lastly, a set of clinical MRI scans matching the postmortem data set was collected by contacting brain donors’ families to find the hospitals where they were treated at the end of their lives and request a copy of their most recent brain MRI scans. Despite our efforts, in vivo brain MRI scans could only be recovered for a minority of the brain donors. Various protocols and scanners were used to produce these MRI scans, but most of the scans were T1-weighted and acquired at low anisotropic resolutions in clinical 1.5 Tesla scanners several years before the donor’s autopsy.

The limited scan resolution of these scans raised significant challenges during their processing, and the time gaps between in vivo and postmortem scans required specific caution during the analysis. By retaining the MRI sessions with either one T1-weighted MRI scan acquired at an isotropic resolution or two or more anisotropic T1-weighted MRI scans, we obtained 35 MRI sessions. These sessions cover 31 of the 92 brain donors since two in vivo MRI sessions were recovered for four brain donors (four men). Table 1 presents the demographics of the 31 brain donors with in vivo data. For the four men with two in vivo MRI sessions, the average of the two ages at scanning time was retained. As in the overall sample of 92 brain donors, no significant age difference was observed between men and women within the sample of in vivo scans. More data was recovered for men, who account for 19 of the 31 sets of in vivo scans (61 %), but this small proportion increase compared to the overall sample was not strong enough to reach significance when conducting a Fisher’s Exact (p-value 0.41).

Table 1 also reports the average time between in vivo and postmortem scans, which will be referred to as age gap. This age gap was close to 3 years and 300 days and very similar between men and women (p-value 0.94, two-sample *t*-test).

Lastly, Table 2 indicates that among the 35 in vivo MRI sessions recovered, only two sessions were made of a single, isotropic T1-weighted MRI scan. Most of the sessions were containing two or three T1-weighted scans acquired in different directions, such as pairs of coronal and axial scans (respectively acquired with a limited resolution in the coronal or the axial direction). In the remaining few sessions, up to seven scans could be exploited to conduct the present analysis.

**Table 2:** Number of in vivo clinical MRI sessions recovered as a function of their number of available T1-weighted scans. A single isotropic MRI scan was available for only two sessions. For most of the sessions, two or three anisotropic MRI scans acquired with different orientations were available.

T1 scans in the session	1	2	3	4	5	7
number of sessions	2	16	11	4	1	1

### 2.6 Segmentation challenges and pipelines

The original study goal was to process antemortem and postmortem data using the same parcellation pipeline combining standard in vivo MRI processing methods. A pipeline in five steps was then designed: MRI intensity correction, skull-stripping, brain tissue segmentation, registration of a set of atlases to project their ROIs onto the antemortem and the postmortem brains, followed by a majority voting fusing the registered atlases into a single parcellation aligned with the segmented brain tissues. The twenty brains of the OASIS-TRT-20 data set (Marcus et al., 2007) annotated according to the Desikan-Killiany-Tourville protocol (Klein and Tourville, 2012) extending the Desikan atlas (Desikan et al., 2006) were selected as atlases. Unfortunately, both the in vivo and the postmortem data raised challenging issues that required conflicting adaptations of this generic approach. We ended up with two distinct pipelines generating their brain masks, their tissues segmentation, and their brain maps via different approaches and different versions of the OASIS-TRT-20 atlases.

### 2.7 Postmortem pipeline

For the postmortem scans, brain masks and tissue segmentations were particularly challenging to obtain because our postmortem samples were imaged within plastic bags filled with formalin. The contrast observed in the T1-weighted images was insufficient to successfully separate grey matter and white matter, so the pipeline was modified to process, segment, and register the T2-weighted MRI scans. Unfortunately, the T2- weighted MRI signal was too strong within the formalin surrounding the brains to resort to simple methods for generating brain masks, such as intensity thresholding. Even for the T1-weighted scans, where the formalin appeared darker than most brain tissues, standard skull-stripping methods such as FSL BET (Jenkinson et al., 2012) failed to provide decent brain masks. Because the scans were too time-consuming to segment manually, it was necessary to develop a new, dedicated brain segmentation approach, where T2-weighted and T1-weighted MRI scans were combined to separate brain tissues from the surrounding formalin. This approach, described in detail in the following subsections, required a resampling and a registration of the T2-weighted and T1- weighted scans acquired for the same brain sample. That registration was significantly more successful when carried out for intensity-corrected, denoised scans. Denoised scans were also necessary for the brain tissue segmentation. Lastly, a visual quality control of the scans revealed that several postmortem samples had been acquired in the wrong position. This issue caused multiple registration failures, so it was decided to check all the scans, manually swap and flip their axes when necessary to re-orient them all in the same manner, and systematically inspect all the steps of the scans processing.

**Fig. 1:**
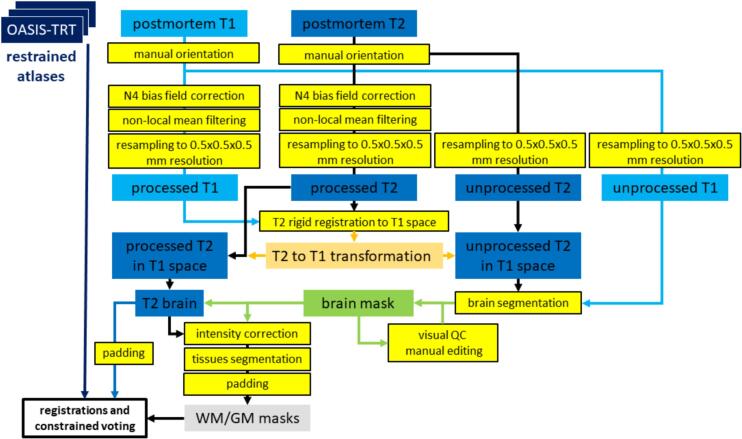
Processing pipeline to parcellate the postmortem samples considered in this work. For each sample, a postmortem T1-weighted and a postmortem T2-weighted MRI scans were available. Both scans were manually re-oriented and either resampled at a spatial resolution of 0.5 mm, or bias field corrected and denoised before the resampling. The processed scans were used to register the two MRI sequences and identify the white matter and the grey matter in the brain (WM/GM masks), whereas the unprocessed scans were used to segment the brain.

Taking all these constraints and issues into account led to the elaboration of the pipeline presented in Fig. 1. In this approach, all the T1-weighted and T2-weighted MRI scans were first visually inspected and manually reoriented. Then, for each brain sample, two versions of the T2-weighted scan were generated: a processed version that was produced by applying the N4 bias field correction (Tustison et al., 2010) provided with the Advanced Normalization Tools software (ANTS (Avants et al., 2011); version 2.2.0) library, followed by the non-local mean filtering NAONLM3D (J. Manj́on et al., 2010); and an unprocessed version that was neither bias field corrected nor denoised. In parallel, the T1-weighted images were processed in the same way to produce an unprocessed T1-weighted image and a processed T1-weighted image via N4 intensity correction and NAONLM3D denoising. All the images were then resampled at an isotropic spatial resolution of 0.5 mm per voxel using ANTS (version 2.2.0) ResampleImage command set for a windowed Sinc interpolator (Avants et al., 2011). ANTS library was used to determine a good rigid transformation to warp the processed T2-weighted image in the space of the processed T1-weighted image. This rigid transform was applied to the unprocessed T2-weighted image. A Deep Learning method was then used to generate a brain mask by jointly considering the unprocessed T1 and the unprocessed T2 warped in the T1 space. This Deep Learning method is described in detail in Section 2.3.1. The brain masks were all visually inspected, manually edited when necessary, and used to mask the processed T2 images registered in the T1 space. The MRI intensity within these T2 brain images was corrected by the method described in Section 2.3.2. The FSL FAST software (version 5.0.11) was used to segment the intensity-corrected brains into three regions: white matter, grey matter, and cerebrospinal fluid, which was masked out (Jenkinson et al., 2012). The white matter map, the grey matter map, and the intensity-corrected T2 brain image were padded with five layers of zeros. Lastly, the registration method described in Section 2.3.3 was used to project all the OASIS-TRT-20 atlas parcellations onto the intensity-corrected T2-weighted brain image, and the constrained majority voting procedure presented in Section 2.3.4 was employed to generate a single, final parcellation for the postmortem sample. This parcellation was finally used to compute the volumes of a set of brain structures of interest, such as the Cerebellum, the cerebrum white matter, the deep grey matter regions, and the hippocampus. When two scans were processed for the same individual, the final volumes were averaged.

### 2.8 Deep postmortem brain segmentation

Failed preliminary tests conducted using FSL BET (Jenkinson et al., 2012) suggested that the segmentation of brain samples in single postmortem MRI scans is particularly challenging for standard skull-stripping tools. Fortunately, we realized that the issue can be addressed by considering the two MRI sequences at the same time: the air outside the formalin bag storing the brain sample appears extremely dark in both T1 and T2 weighted scans, while the formalin appears darker than most brain tissues in T1 scans and significantly brighter in T2 scans, and these intensity changes are very distinct from the changes observed in brain tissues. The textures are also strikingly different: contrary to brain tissues where millimeter-small details are visible, the formalin usually presents a very smooth signal only altered by magnetic field and reconstruction artifacts producing centimeter-wide fluctuations. It was then possible to distinguish the brain tissues from the background by modeling multimodal texture patterns.

**Fig. 2:**
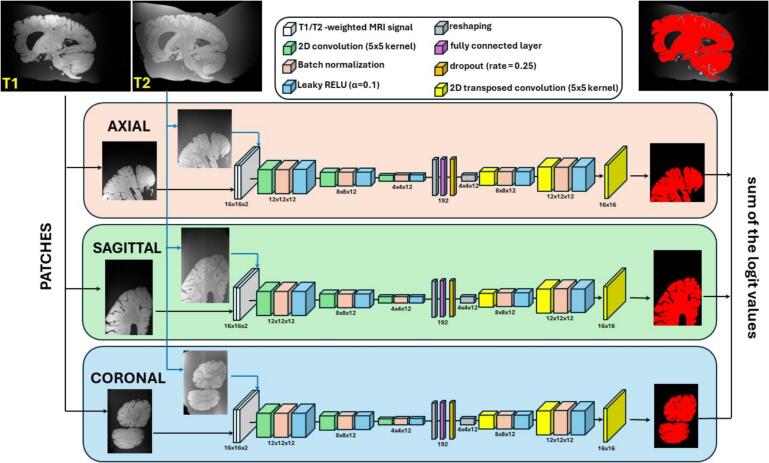
Deep Learning approach adopted in this work to segment the postmortem brain samples: pairs of patches extracted every 5 voxels from the T1 and the T2-weighted MRI scans were segmented by three autoencoders respectively trained to segment axial, sagittal, and coronal pairs of patches. The logit values produced by the three autoencoders were summed over the overlapping patches and the final brain mask was obtained by retaining voxels with a positive sum of logits.

Deep Networks are perfectly fit for this task because they can combine multiple channels of information, and they have achieved outstanding performance in binary segmentation tasks due to their ability to learn intricate texture patterns (Ronneberger et al., 2015). In this work, three small autoencoder architectures were trained: a model segmenting 2D patches of size 16x16 extracted in the axial direction, a model segmenting sagittal patches of the same size, and a model segmenting coronal patches. The patches were simultaneously extracted from the unprocessed T1-weighted and the unprocessed T2-weighted scans registered in the T1 space and concatenated to create 2-channel input images. The models were then applying three convolutional layers (Krizhevsky et al., 2012); each layer applying twelve 5x5 convolution kernels, a batch normalization (Ioffe et al., 2015); and a leaky RELU with parameter *α* = 0.1 (Nair and Hinton, 2010). The 192 output features were flattened, a fully convolutional layer with the same 192 output size was applied (Krizhevsky et al., 2012) followed by a dropout layer with rate 0.25 (Srivastava et al., 2014); and the 192 features were reshaped and subjected to three deconvolutional layers with the same structure than the convolutional layers (twelve 5x5 kernels, batch normalization, leaky RELU). In the last layer, logits were produced by applying no batch normalization and no RELU transformation. The last layer was designed to apply a single 5x5 kernel instead of twelve to output 16x16 patches of real values. Fig. 2 presents these architectures in detail. Overall, each model only had 52,657 parameters: 120 non-trainable parameters and 52,537 trainable parameters. These relatively compact models could be optimized by Deep Learning libraries running on the CPUs of a standard office computer.

Two sets of experiments were conducted. A cross-validation was first carried out with ten manually segmented pairs of postmortem scans to estimate the quality of the models. Then, the entire set of data prepared for the crosss-validation was used to train a final triplet of models, that was applied to the remaining 85 pairs of scans. The 85 segmentations were all visually inspected and manually edited when necessary.

More specifically, the models were implemented using Keras/Tensorflow libraries. All the models were trained using Adam optimize with parameter *β*_1_ = 0.9, *β*_2_ = 0.999, and using amsgrad. The batch size was fixed to 250 and 25 training epochs were repeated for 5 decreasing values of learning rate: 0.02, 0.01, 0.005, 0.002, 0.001 (125 epochs in total) to optimize a binary cross-entropy loss calculated from logits. During the cross-validation 25,000 pairs of patches were extracted at random locations within each annotated pair of scans, independently for each model direction and together with the corresponding patches of manual segmentation labels. For each pair of postmortem scans, a triplet of models was trained using the patches of the nine remaining pairs of scans (225000 patches for each model). The models were tested by extracting for both sequences and each direction a patch at every voxel in that direction but every over 5 voxels in the orthogonal directions (to achieve a 25-fold speedup during the testing), applying the model correspond- ing to the direction of each pair of patches, summing the logits produced by the three models over all patches/directions, and selecting voxels with positive sums. A set of morpho-mathematical operations was then conducted to clean the final mask: filling the holes in the mask and removing the disconnected clusters of voxels. These final masks were compared with the manual annotations by computing Dice overlaps (Dice, 1945); true positive and negative rates. Once the cross-validation completed, the 250,000 training patches extracted from the ten training pairs of scans in each direction were used to train a final triplet of models that were applied to the remaining unlabeled 85 pairs of MRI scans in the same way as the test images (5 voxels stride overlapping patches, thresholded sum of logits, morpho-mathematical operations).

### 2.9 Intensity correction

After observing that the original N4 bias field correction applied to the raw T2-weighted postmortem scan was unable to remove all the magnetic field inhomogeneity signal artifacts, a second step of signal intensity correction was introduced in the pipeline. This intensity correction was applied after masking out the formalin and the background of the processed T2-weighted scans warped in the space of the postmortem T1-weighted scans, and used a Gaussian smoothing kernel to cut low spatial frequency components in the T2-weighted images.

More specifically, the intensity correction was carried out in five steps. First, the masked T2-weighted scans were convolved with a Gaussian smoothing kernel. The corresponding binary brain masks were smoothed in the same way, to produce a set of smoothed masks taking continuous values. The smoothed T2-weighted signal was then divided by the values in the smoothed mask, and the result was restricted to the brain by multiplying by the un-smoothed mask. Lastly, the output was multiplied by a scalar to set the mean intensity after intensity correction at the same value as the signal mean before correction. During our experiments, the Gaussian smoothing standard deviation was set to 2 mm to remove magnetic field artifacts and attenuate large pathological structures, such as widespread white matter lesions, without altering the contrast at the borders between the white matter and the grey matter.

### 2.10 Registrations with restricted atlases

In the protocol currently implemented at the South Texas Alzheimer’s Disease Research Center Post- mortem Repository, the brain samples are separated in two parts: the left hemispheres are scanned before dissection while the right hemispheres are frozen for a long-term storage (Li et al., 2023). This dissection protocol re- quired the use of two different types of atlases: the original OASIS atlases registered in the MNI152 space and covering the entire brain were used for the parcellation of in vivo scans, while a new set of 20 atlases restricted to the left hemisphere was used to process the postmortem scans. These restricted atlases were derived as follows. First, the brain regions labeled as ventricles were removed from the OASIS atlases registered in the MNI152 space. All the labels corresponding to brain regions part of the right hemisphere and the right cerebellum were removed. Lastly, the brain regions covering both hemispheres were cut by erasing labels and T1 signal for all the voxels on the right of the brain midline, which is precisely aligned with the abscissa x = 90 for the MNI152 templates used to register the OASIS scans (Marcus et al., 2007).

An anatomical specificity of the postmortem brains required an additional adaptation of the restricted atlases. Specifically, in postmortem samples, the cerebellum floats in the fixation fluid several millimeters away from the forebrain. Since most registration methods rely on and enforce the assumption that images should not be torn apart during their registration, brain maps derived from in vivo scans where the cerebel- lum and the forebrain are in contact, as in the OASIS brains, are doomed to produce wrong postmortem parcellations, where either cerebellum or cortical labels will be warped to the wrong structure or cover the fixation fluid between them. During our experiments, this issue was addressed by eroding the postmortem atlases: in the OASIS atlases restricted to the left hemisphere, all the cerebellum voxels within two voxels of brain voxels labeled with cortical labels were erased. This erosion opened a thin space between the cor- tex and the cerebellum that could be inflated by the registration algorithms in their attempt to catch the cerebellums floating away from the forebrains. This simple trick significantly improved the segmentation of the cerebellar regions during our experiments.

The choice and the derivation of the restricted atlases required by our postmortem brain samples con– stitutes the most innovative part of our multi-atlas registration approach. The registrations between the atlases and the brains segmented in the denoised T2-weighted postmortem scans were, on the opposite, ob- tained via one the most well-established and robust registration method: the SyN registration method (Avants et al., 2008) implemented in the Advanced Normalization Tools software library (ANTS (Avants et al., 2011); version 2.2.0), which was set to calculate mutual information metrics when warping the restricted atlases derived from T1-weighted scans to the well-contrasted T2-weighted postmortem scans.

### 2.11 Tissue-constrained majority voting

Despite the use of restricted atlases and robust registration methods, a significant number of registrations failed to align the OASIS-TRT-20 atlases with the postmortem scans. Small warping errors were observed in almost all samples and would have blurred the frontiers between grey matter and white matter if a straightforward majority voting had been applied, resulting in a significant number of white matter voxels to be parcellated with atlas labels corresponding either to cortical or deep grey matter regions. We also observed that the OASIS-TRT-20 cerebellum masks were lacking the level of detail observed in our postmortem scans, where multiple thin white matter regions could be distinguished from the surrounding grey matter for most brain samples. Even if the registrations were perfect, an accurate segmentation of the cerebellum in the postmortem scans was then impossible to obtain without exploiting an additional source of information providing the location of the brain tissues to refine the atlas labels.

These conclusions justified the development of an advanced tissues-constrained majority voting scheme relying on the brain tissues maps generated from the processed postmortem T2-weighted scan (see Fig. 1). More specifically, the regions of the OASIS-TRT-20 atlases were listed and grouped into white matter regions, grey matter regions, and ventricle/CSF regions that were removed from the final parcellations (Desikan et al., 2006, Klein and Tourville, 2012, Marcus et al., 2007). The twenty OASIS-TRT-20 parcellations warped into each individual processed T2-weighted postmortem scan were then combined in two steps.

In the first step, each warped parcellation was cleaned according to the tissue maps generated for the corresponding brain sample: the atlas labels corresponding to white matter regions were removed from voxels segmented as part of the grey matter, and grey matter labels were removed from the white matter tissue. Cerebellum labels were fixed by swapping grey and white matter label values to match cerebellum tissues. Then, isolated groups of grey matter and white matter voxels were removed by running a connected component analysis independently for each atlas label, except for the cerebellum labels that were, at that step, often aggregated into fragmented clusters and required specific processing. The cerebellum white matter labeling was fixed by running a region growing algorithm marking at each step the unlabeled white matter voxels within a 3 x 3 x 3 neighborhood of a white matter cerebellum voxel as white matter cerebellum tissue. Lastly, the cerebellum grey matter mask was improved by marking all the unlabeled grey matter voxels with strictly more than five white matter cerebellum voxels in their 7 x 7 x 7 vicinity as cerebellum grey matter tissue. This 7 x 7 x 7 vicinity was chosen to counteract the atlas erosion applied when preparing the restricted atlases.

Once the individual parcellations ready, they were combined by a majority voting scheme using the brain tissue maps a second time to prevent an alteration of tissues borders. More specifically, the majority voting scheme proceeded first with the white matter: for each white matter voxel, when possible, the twenty parcellations were checked and the most frequent white matter label at that location was retained. Disconnected white matter components were removed, and a region-growing algorithm was run to gradually select labels for the white matter voxels without labels. The labels were selected by finding the most frequent white matter labels already selected in the 7 x 7 x 7 vicinity of the voxels to label. Lastly, the isolated clusters of white matter voxels that could not be reached by the region-growing algorithm were considered tissue segmentation failures and re-assigned to the grey matter. Once the white matter parcellation obtained, the grey matter parcellation was derived by majority voting. Due to the larger number of grey matter labels and the frequent registration errors, spatial smoothing was necessary during the majority voting.

The majority voting used for the white matter was then adapted to take all the grey matter labels in the twenty registered parcellations within a 5 x 5 x 5 neighborhood into account (up to 2500 label values, then). The voxels within the neighborhood were weighed by a Gaussian function of the Euclidean distance to the center of the neighborhood with sigma √2. These neighborhood size and smoothing parameters were manually selected to prevent over-smoothing while maintaining significant smoothing effects. Lastly and since the cerebellum grey matter masks of the cleaned parcellations were already satisfying, grey matter voxels selected as cerebellar grey matter in more than a quarter of the cleaned parcellation were directly set as cerebellar grey matter in the final parcellation. This step imposed a straightforward majority voting for the cerebellum grey matter instead of the voting scheme with spatial smoothing applied to the cortical and deep grey matter regions and produced better results in the cerebellum.

### 2.12 In vivo pipeline

Most of the time, when an in vivo scan was available for a brain donor, it was a T1-weighted MRI scan acquired with a limited and anisotropic spatial resolution: the brain was acquired with a slice thickness much larger than the spatial resolution inside the slices. These limited spatial resolutions could have raised serious issues during scan processing and, particularly, the estimation of the brain regional volumes. Fortunately, the brains were usually scanned multiple times at different orientations during the same clinical session. A simple four-step super-resolution procedure was then developed to combine the different orientations and fix the spatial resolution. The mri synthsr method (Iglesias et al., 2021, Iglesias et al., 0000) provided with the Freesurfer library (Dale et al., 1999); version 7.4.0) was first used to synthesize 1 mm isotropic resolution images close to the original scans. ANTS rigid registration was then used to register all the MRI volumes to the first one in the session (Avants et al., 2011); version 2.2.0). Since mri synthsr tends to modify the MRI intensity of a scan, an ad-hoc non-linear intensity matching procedure was used to align the MRI signal intensity of the synthesized scans with the intensity distribution of the first synthesized scan, that consists in: (i) randomly sampling, sorting, and grouping by batch of 29 values a set of 100,000 voxel intensity values in the source scan to modify, (ii) calculate for each batch the median intensity value in the corresponding set of 29 values sampled at the same locations in the target scan,

(iii) find for each batch the source intensity associated with the target intensity value which is the closest to that batch median value to create a mapping, and (iv) apply the mappings to the entire source image to generate a new image with a similar intensity distribution as the target. After matching their intensity, the registered synthesized scans were finally averaged to produce a single isotropic T1 image combining all the available scans.

**Fig. 3:**
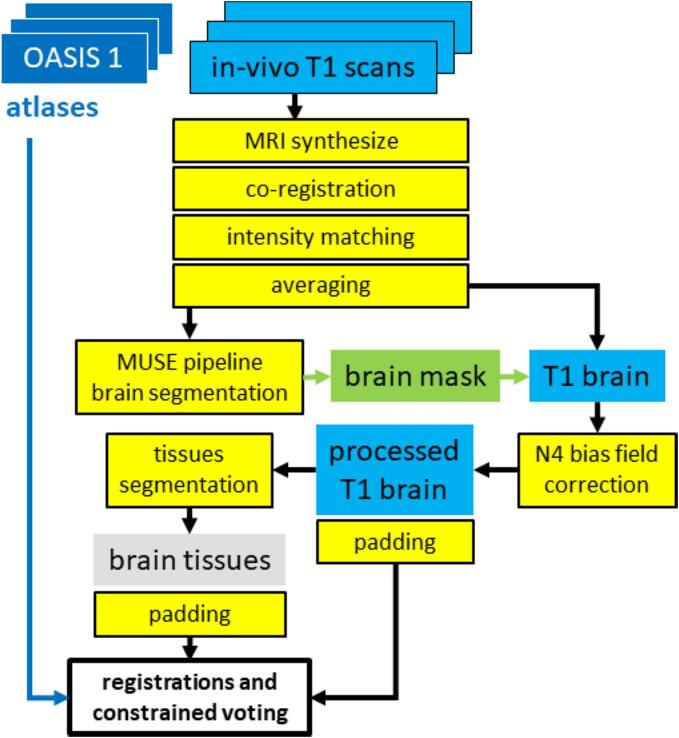
In vivo data processing. Contrary to the postmortem samples, only T1-weighted MRI scans of limited quality were available, but several scans were recorded for each brain. A super-resolution approach was then adopted to generate an average brain volume of isotropic 1 *mm ×* 1 *mm ×* 1 *mm* resolution, that was then processed to extract a brain mask, segment tissues, and register the atlases.

Contrary to the images of the postmortem samples, the averaged in vivo scans could be processed using standard methods and atlases. The multi-atlas MUSE pipeline was then used to skull-strip the brain (Doshi et al., 2016), the N4 bias field correction (Tustison et al., 2010) to remove scanning artifacts prior to image registrations, and the SyN registration method (Avants et al., 2008) provided with ANTS (Avants et al., 2011); version 2.2.0) was used to register the twenty original OASIS-TRT-20 atlases in the space of the individual brains (Desikan et al., 2006, Klein and Tourville, 2012, Marcus et al., 2007). Lastly, the FSL FAST method (Jenkinson et al., 2012), version 5.0.11) was used to derive brain tissue maps, and the constrained majority voting method developed for the postmortem scans was applied to combine the twenty parcellations into a final brain map. The complete procedure is presented in Fig. 3. When two scans were processed for the same individual, the final volumes were averaged.

### 2.13 Voronoi Tessellations and consensus atlas

In the OASIS-TRT-20 atlases, the white matter inside the cerebrum is indicated as a single region of interest (Marcus et al., 2007, Klein and Tourville, 2012). This labeling is not sufficient to localize brain alterations happening in the white matter, such as white matter hyperintensities. During our experiments, this issue was addressed by conducting a Voronoi tessellation of the final cerebrum white matter region generated by our multi-atlas registration pipeline according to the neighboring grey matter regions of interest. More specifically, each voxel labeled as a cerebrum white matter location was assigned a new label indicating its closest grey matter region of interest in terms of Manhattan distance (L1 distance). This post-processing step parcellated the white matter into subcortical regions that were measured to quantify white matter volume changes with more precision.

**Fig. 4:**
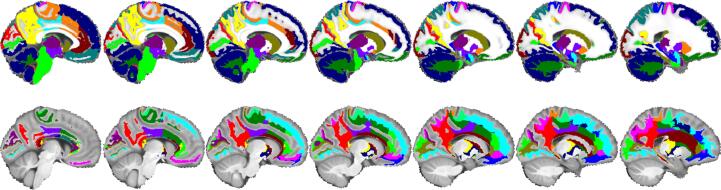
Consensus atlas. Top: OASIS-TRT-20 labels without cerebrum WM. Bottom: cerebrum WM Voronoi tessellation.

Lastly, a consensus atlas was generated by registering all the OASIS-TRT-20 atlases in the space of the Conte69 atlas (Van Essen et al., 1991) and applying the majority voting method to create a single, clean parcellation in that high-resolution atlas. The atlas was then split into halves to only retain the left hemisphere. All the statistical finding derived for specific brain regions of interest will be visualized in that atlas space. Like the individual parcellations, the consensus atlas white matter parcellation was also refined by conducting a Voronoi tessellation of its white matter cerebrum region. Fig. 4 presents the consensus atlas and its parcellations.

### 2.14 Implementation details

All the statistical tests were implemented in R, version 4.1.1 (R Core Team and R: a, 2021), except for linear models that were fit using the Python statsmodels API (version 0.12.2) set to conduct robust linear regression using a robust norm based on Huber’s estimator (Huber, 1964). Spearman rank correlations and their p-values were also calculated in Python using the Scipy library (version 1.6.2). All the image processing pipelines developed in this work were containerized. However, contrary to most neuroimaging processing pipelines released so far that are packed in single, large containers (Craddock et al., 2013, Esteban et al., 2019, Waller et al., 2022); a modular approach was adopted in this work by embedding the required image processing libraries in small separate Docker containers that were only accessed when needed (Docker, 0000). Computing performances were further boosted by reforming the Docker containers into compact Singularity containers that required up to five times less random-access memory (RAM) and ran faster than their original counterparts (Kurtzer et al., 2017).

## 3 Results

### 3.1 Brain segmentation

For each pair of scans included in the cross-validation experiments, the mask generated by our triplet of autoencoders was compared with the manual annotations by computing a Dice overlap (Dice, 1945); a true positive rate (TPR) and a true negative rate (TNR). More specifically, the number of true positive voxels (TP voxels automatically segmented as part of the brain that were manually annotated as part of the brain), true negative voxels (TN voxels segmented and annotated as background), false positive voxels (FP voxels segmented as brain voxels while annotated as background) and false negative voxels (FN voxels segmented as background while annotate as brain voxels) were counted and the following formulas were applied:

(1) $$\mathrm{Dice}\;=\frac{2TP}{2TP+FP+FN}$$

(2) $$\mathrm{TPR}=\frac{TP}{TP+FN}$$

(3) $$\mathrm{TNR}=\frac{TN}{TN+FP}$$

Table 3 reports the minimum, maximum, and average metric values measured when testing the ten pairs of postmortem MRI scans, as well as pulled metrics obtained when summing the counts of voxels over the ten annotated brain samples. The Deep Networks produced almost perfect segmentations: on average, 97.4 % of the brain voxels were correctly segmented by the models (TPR), and 99.52 % of the background voxels were discarded from the brain masks (TNR). These results correspond to an average Dice of 97.57 %. The pulled metrics are almost identical. Interestingly, the minimum metric values are also very high, which indicates that none of the ten triplets of models failed to produce a very accurate segmentation.

**Table 3:** Segmentation metrics measured during the cross-validation of the Deep Network models used in this work to segment the postmortem brain samples. The pulled metrics were computed by adding the counts of true positive, false positive, true negative, and false positive voxels across the ten testing pairs of scans.

metric	average	min	max	pulled
Dice	0.9757	0.9685	0.9845	0.9759
TPR	0.9740	0.9546	0.9880	0.9753
TNR	0.9952	0.9893	0.9988	0.9954

These results were considered to be sufficiently good to process the 85 remaining pairs of scans, and instead of conducting further segmentation experiments, the time was spent manually correcting the 85 brain masks automatically segmented. Overall, the set of 95 brain masks required a couple of weeks to manually segment the first 10 brain masks from scratch, a couple of weeks to carry out the cross-validation experiment and the final model training, and a few hours of manual editing to fix the few mistakes observed in the 85 masks automatically generated by the models.

### 3.2 White matter hyperintensity correction

**Fig. 5:**
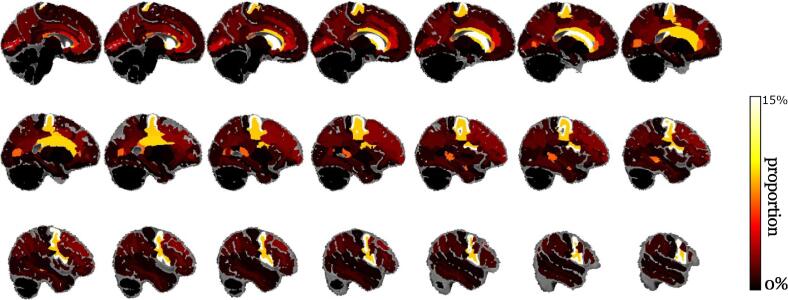
Proportion of brain regions covered by white matter hyperintensities (mean over the 42 brain donors with masks).

For the forty-two brain donors with hyperintensity masks, the volume of white matter hyperintensities was measured within each brain region and divided by the volume of the brain region. The mean over the forty-two brain donors of the fraction measured for each brain region is reported in Fig. 5. The putamen was the region containing the most hyperintensities (17.52 %), followed by the precentral cortical region (14.61 %), the Voronoi white matter region close to the putamen (9.21 %), the Voronoi white matter region close to the precentral cortical region (8.96 %), and the anterior corpus callosum (8.53 %).

**Fig. 6:**
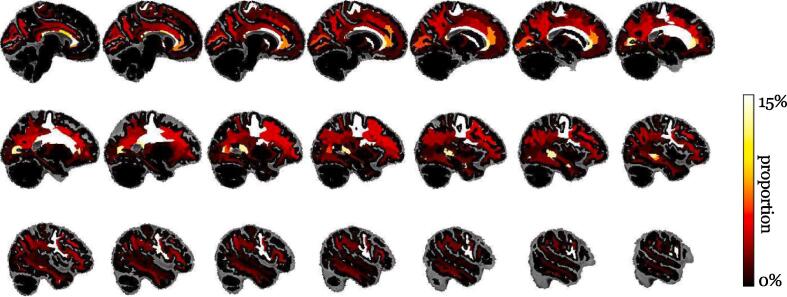
Mean proportions of white matter hyperintensities after the corrective swap.

Many regions are grey matter regions that are not supposed to contain white matter voxels and should then contain no white matter hyperintensities. This issue certainly happened because we had to consider the T2-weighted postmortem scans, where white matter voxels with hyperintensities closely resemble grey matter voxels, to derive the brain tissue maps guiding our parcellations. During the following experiments, this issue was mitigated by swapping the volumes of white matter hyperintensities within a grey matter region between that region and the closest white matter sub-region defined by the Voronoi tessellation. This corrective swap produced the fractions shown in Fig. 6: the white matter region with the most hyperintensities is still the Voronoi region associated with the putamen with an average of 28.14 % of hyperintensity now, the Voronoi region associated with the anterior corpus callosum (19.66 %), the Voronoi subcortical region associated with the precentral cortical region (17.93 %), the white matter close to the choroid plexus (13.00 %), and the white matter region close to the mid anterior corpus callosum (10.26 %).

The imbalance of the white matter hyperintensity volumes between men and women was assessed by conducting Wilcoxon rank-sum tests for each Voronoi region separately. None of these statistical tests was significant at level 0.05 after a Bonferonni correction for the 46 Voronoi regions, but the volume of white matter hyperintensities in the entire cerebrum white matter was very significantly lower for women (median volumes 5521 *mm*^3^ for women, 16044 *mm*^3^ for men, p-value 4.0 x 10^-4^). Similarly, the Spearman rank correlation between the white matter hyperintensity volume and the age at death of the brain donor was calculated for each region to examine the increase of hyperintensities with age. None of the correlations was significant at a level of 0.05 after the Bonferonni correction. The total volume of white matter hyperintensities was not significantly correlated with age (*ρ* = 0.133, p-value 0.40), even when the correlation was separately calculate for men (*ρ* = 0.169, p-value 0.45) and women (*ρ* = 0.09, p-value 0.72).

According to these statistical results, the regional volumes of the 50 brain donors without manually delineated white matter hyperintensity masks were only corrected based on their gender: for each grey matter brain region, a volume swap between that brain region and the closest Voronoi white matter sub- region was conducted for a volume corresponding to the average of the volumes swapped for the brain donors with a hyperintensity mask and the same gender. Lastly, the overall volume changes conducted on a postmortem brain sample were replicated for the available in vivo regional volumes.

### 3.3 Morphometry

After correcting the regional volumes for the presence of white matter hyperintensities, it was finally possible to conduct the morphometry analysis. This analysis was carried out to achieve four goals: check the presence of significant age effects and, in particular, significant atrophy in the brain regions known to be impacted by Alzheimer’s disease, compare the regional volumes estimated in the in vivo clinical scans with the postmortem regional volumes, keep the additional covariates into account, and investigate the relation between regional volumes and the clinical diagnosis. These four goals were achieved at once by taking both in vivo and postmortem regional volumes into account in a single robust linear model, predicting the regional volume by combining the age of the brain donor at the time of the scan, their sex, their diagnosis, and a volume shift only applied to the in vivo scans (*invivo* is a binary variable equal to one if and only if the volume was derived from an in vivo clinical scan):

(4) vol.∝α+β.age+γ.sex+δ.invivo+ηD

where the diagnosis *D* was encoded using a set of binary variables, each variable corresponding to the presence of a dementia:

(5) $$\eta D=\eta_{1}AD+\eta_{2}FTD+\eta_{3}OD+\eta_{4}VD+\eta_{5}M$$

The binary variable *M* was set to 1 for the fifteen missing diagnosis. For these brain donors, the other binary variables were set to 0. The clinical diagnosis of the in vivo scans was copied from their associated postmortem diagnosis, assuming that the time between in vivo and postmortem scans was too short to correspond to the development of a dementia.

Combining in vivo and postmortem volumes moderately increased the sample size, up to 123 values per linear model, increasing the likelihood of detecting small effects as significant. But if a linear model was fitted for each small brain region defined in the atlas parcellation, the effects would probably be discarded by a Bonferonni statistical correction for multiple comparisons. The analysis was then focused on five large interesting brain areas: the total brain volume, the whole cerebrum white matter volume, the deep gray matter volume, the volume of the hippocampus, and the cerebellum volume.

*Total brain volume.* The robust linear model derived for the entire brain volume detected no significant volume decrease with aging. On the opposite, women’s brains appeared significantly smaller, of 75200 mm^3^, which corresponds to 15.6 % of the average volume (483400 cubic millimeters). The brains extracted from in vivo clinical scans were also significantly larger (by 69600 mm^3^, 14.4 %).

*Cerebrum white matter.* For the cerebrum white matter, the observations were the same for sex differences (smaller cerebrum white matter by 41600 mm^3^ for women, which corresponds to 20.0 % of the overall average volume). However, no significant difference in volumes could be established between in vivo and postmortem parcellations.

*Deep grey matter.* The volume of the deep grey matter was measured by summing the volumes of the thala- mus, caudate, putamen, pallidum, hippocampus, amygdala, and accumbens area (labels 10,11,12,13,17,18,26 in the atlas (Klein and Tourville, 2012). Like the previous regions, the deep grey matter was significantly smaller for women (of 1900 mm^3^, which corresponds to 11.3 % of the average deep grey matter volume). A significant atrophy was associated with age (105 mm^3^ per year, p-value 3.54 x 10^-4^). The volumes derived from in vivo scans were significantly smaller, of 2120 mm^3^ (12.6 %, p-value 9.32 x 10^-4^). A diagnosis of frontotemporal dementia (FTD) was also associated with smaller volumes: on average, 3020 mm^3^ (17.9 %), but the p-value of 0.0255 would not pass a Bonferroni correction for five regions.

*Hippocampus.* The model focusing on the hippocampus volume revealed that the brain structure was im- pacted by the largest number of effects: in addition to significant differences associated with sex, age, and frontotemporal dementia, the structure was also affected by Alzheimer’s disease. A diagnosis of AD was associated with a reduction of the hippocampus of 425 mm^3^ (13.0 %), but the p-value of 0.0388 does not pass the Bonferroni correction. Contrary to the other deep grey matter regions that appear larger in postmortem scans, however, the hippocampal volumes estimated for in vivo scans were, on average, larger by 42 %.

*Cerebellum.* For the total cerebellum volume, obtained by summing cerebellar grey and white matter vol- umes (labels 7 and 8 in the atlas (Klein and Tourville, 2012), on the opposite, no significant dementia effect was observed. Smaller volumes were reported for women, and an age-related atrophy is present. In addition, the volumes derived from in vivo scans are significantly smaller by 3670 mm^3^ (6.7 %). These results are summarized in the Table 5.

### 3.4 Neuropathology scores

The scores were missing for a brain donor who was then removed from the analysis. Table 4 reports linear regressions conducted to evaluate the evolution of the three scores and their sum as a function of the brain donor demographics. No significant difference was observed between men and women, but the Thal phase, the Braak score, and the sum of the three neuropathology scores are significantly increasing with the age at death of the brain donor. More specifically, the results indicate that the brain donors had progressed to a new Thal phase every 18.5 years. On average, they progressed by a Braak stage every twenty years.

**Table 4:** Robust linear models capturing the evolution of the neuropathology scores and their sum with age and sex.

neuropathology score *∝* cst. + sex + age				
**neuropathology**		**cst.**	**sex**	**age**
Thal		0.052	-0.381	0.054
	p-value	0.964	0.202	**0.00019**
Braak		1.013	−0.230	0.050
	p-value	0.423	0.484	**0.00168**
CERAD		−0.238	−0.173	0.029
	p-value	0.796	0.473	0.01286
sum of the scores		−0.384	−0.772	0.146
	p-value	0.910	0.383	**0.00065**

The neuropathology scores are highly correlated. More specifically, we measured a Spearman rank correlation of 0.703 between the Thal phase and the Braak stage (p-value 7.64 x 10^-15^), of 0.764 between the Thal phase and the CERAD score (p-value 1.26 x 10^-18^), and of 0.781 between the Braak stage and the CERAD score (p-value 6.25 x 10^-20^). To avoid collinearity issues when conducting regressions, the individual scores were then replaced by their sum.

**Fig. 7:**
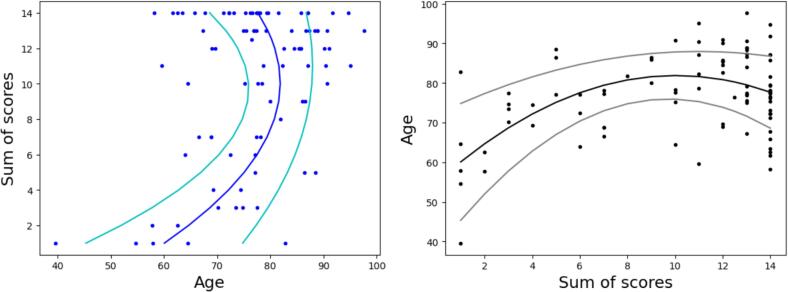
On the left: sum of neuropathology scores as a function of age. On the right: age as a function of the sum of neuropathology scores. The curves present the robust quadratic fit of the age as a function of the sum of neuropathology scores (please refer to Section 3.4 for details).

The relation between age and neuropathology scores in our sample is not straightforward. As shown in Fig. 7, the presence of many young brain donors with large neuropathology scores prevents a prediction of the neuropathology scores from the age. On the opposite, it seems possible to model the age of the brain donors as a function of their sum of cognitive scores. The robust quadratic trends displayed in Fig. 7 were obtained in two steps. First, a robust model was fitted to predict the age as a function of the sum of scores (s) and the square of the sum of the scores:

(6) $$\mathrm{age}\propto \alpha +\beta .s+\gamma .s^{2}$$

Then, the same regression was repeated to fit the absolute error of the age prediction:

(7) $$\left|\mathrm{age}-predicted\right|\propto \delta +\eta .s+\lambda .s^{2}$$

(8) $$\text{where }predicted\;=\;\alpha +\beta .s+\gamma .s^{\text{2}}$$

The age and the error predictions were summed and subtracted to visualize a spread around the age pre- diction modeling the absolute error around that prediction.

### 3.5 Neuropathology scores and brain volumes

The nonlinear relation between age and pathology scores also raised challenges to model the relations between regional brain volumes, highly dependent on age, and the neuropathology scores. It was finally decided to model them together by including one of the brain regional volumes as an additional variable in the robust model predicting age as a function of neuropathology scores. More specifically, robust linear regressions of the form:

*age ∝ α* + *β.sex* + *γ.volume* (9)

were conducted and indicated that the deep grey matter (dGM) is the brain region with a volume that is the most significantly associated with age after correcting for sex differences (uncorrected p-value 0.017). The following robust regression was then carried out to associate brain volumes, sex, and neuropathology scores in a single model:

(10) $$\mathrm{age}\propto \alpha +\beta .sex+\gamma .dGM+\eta_{1}.s+\eta_{1}.s^{2}$$

**Fig. 8:**
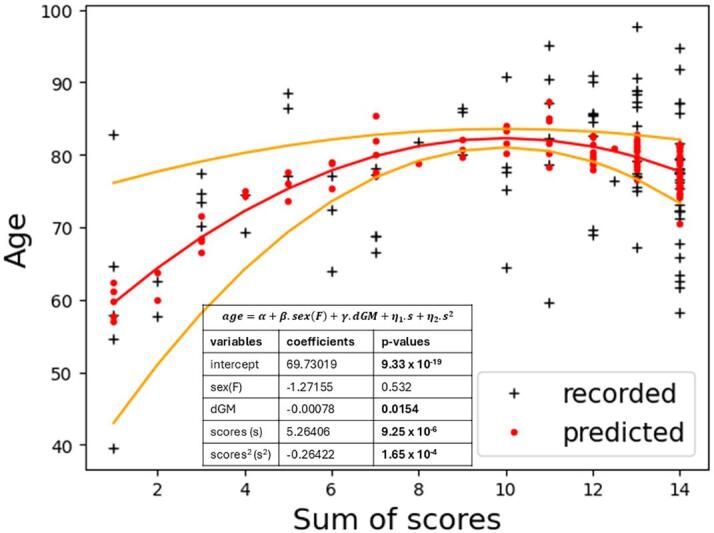
Robust quadratic model fitted to jointly model brain donor demographics (*sex*(*F*) indicates when the brain donor was a woman), the sum of the neuropathology scores (*scores/s*), and the volume of the deep grey matter region (*dGM*). This brain region volume was the most correlated with age. Brain donor age (recorded) and age prediction by the model (predicted) as a function of the sum of neuropathology scores. A quadratic fit was carried out for the age prediction as explained in Section 3.4.

The model obtained, reported in Fig. 8, indicates that the sex differences were not significant. On the opposite, including the volume of the deep grey matter region in the model significantly improved the age prediction. The sign of the coefficients indicates that the age was significantly decreasing with the volume of the deep grey matter. Please note that the statistical models (9) and (10) and the results presented in Fig. 8 do not correspond to a standard morphometric study because they model the age at death as a function of the other variables, while standard morphometric models usually express brain volumes as a function of neurological and biological factors. We decided to explore the statistical models (9) and (10) after noticing that the inclusion of neuropathology scores in the standard morphometric models presented in the previous Section (Table 5) was producing no additional significant effects because of the strong nonlinear relationship between these scores and the age at death (shown in Fig. 8).

**Table 5:** Significant coefficients and associated p-values obtained for all the linear morphometry models. The volume in cubic millimeters of each brain region was modeled as a linear combination of an intercept, a binary variable *sex*(*F*) equal to one for women, the age at scan time, a binary variable equal to one for in vivo scans, and the five binary variables encoding the diagnosis (AD, FTD, OD, VD, M). Five brain regions were considered: the whole brain (Brain), the cerebrum white matter (WM), the deep grey matter (Deep GM), the hippocampus (Hipp.), and the cerebellum (Cerebellum).

**Regression**	volume *∝* intercept + sex(F) + age + in vivo + diagnosis					
volume	coefficients (**uncorrected p-value** *<* **0**.**05)**					
**Brain**						**mean volume**: 4.834 × 10^5^ mm^3^
variable	intercept	sex	in vivo			
coefficient	5.85 × 10^5^	*−*7.52 × 10^4^	6.96 × 10^4^			
p-value	4.45 × 10^−40^	7.10 × 10^−13^	4.00 × 10^-9^			
**WM**						**mean volume**: 2.079 × 10^5^ mm^3^
variable	intercept	sex				
coefficient	2.53 × 10^5^	*−*4.16 × 10^4^				
p-value	7.96 × 10^-27^	1.03 × 10^-13^				
**Deep GM**						**mean volume**: 1.688 × 10^4^ mm^3^
variable	intercept	sex	age	in vivo	FTD	
coefficient	2.66 × 10^4^	*−*1.90 × 10^3^	*−*1.05 × 10^2^	*−*2.12 × 10^3^	*−* 3.02 × 10^3^	
p-value	9.44 × 10^-29^	7.76 × 10^-4^	3.54 × 10^-4^	9.32 × 10^-4^	2.55 × 10^-2^	
**Hipp.**						**mean volume**: 3.276 × 10^3^ mm^3^
variable	intercept	sex	age	in vivo	AD	FTD
coefficient	4.87 × 10^3^	*−*3.88 × 10^2^	*−*1.65 × 10^1^	1.39 × 10^3^	*−* 4.25 × 10^2^	*−* 1.10 × 10^3^
p-value	4.03 × 10^-23^	8.79 × 10^-4^	6.06 × 10^-3^	5.15 × 10^-26^	3.88 × 10^-2^	8.27 × 10^-5^
**Cerebellum**						**mean volume**: 5.480 × 10^4^ mm^3^
variable	intercept	sex	age	in vivo		
coefficient	7.07 × 10^4^	*−*7.65 × 10^3^	*−*1.91 × 10^2^	*−*3.67 × 10^3^		
p-value	4.24 × 10^-38^	4.06 × 10^-9^	4.42 × 10^-3^	1.25 × 10^-2^		

## 4 Discussion

### 4.1 Overview

In this work, we report the findings of a multi-atlas-based morphometry analysis of 92 left brain hemi- spheres donated to the South Texas Alzheimer’s Disease Research Center repository (Li et al., 2023). The in vivo scans available for 31 brain donors were also included in the work, as well as 42 manually delineated white matter hyperintensity masks. In addition, three neuropathology scores could be estimated by dissecting 91 of these brains after their postmortem MRI scans. The postmortem scan settings raised a set of difficulties that are rarely encountered in in vivo clinical studies, such as the need for dedicated algorithms to separate brain samples from surrounding formalin in MRI images and the need to address the insufficient quality of brain scans extracted from medical records. Fortunately, we establish in this work that the combination of multiple MRI sequences and the implementation of cutting-edge Deep Learning methods can address most issues.

### 4.2 Implementation

Our image processing pipelines were containerized. However, contrary to previous image processing pipelines that are usually embedded in single containers (Craddock et al., 2013, Esteban et al., 2019, Waller et al., 2022), we wrapped each image processing library in a single small container that was only used when required (Docker, 0000, Kurtzer et al., 2017). This approach offers the possibility to use different versions of a library within the same pipeline but also, and more importantly, relies on a different programming philosophy: while a single large processing container is used to replicate an entire operating system environment equipped with all the necessary libraries, and will be used to execute advanced Bash scripts calling multiple libraries, our small containers were only used as wrappers for atomic processing steps. This new approach involves more Bash scripting because each processing step now requires to be individually wrapped within a specific container, but the approach grants a tremendous reduction of memory usage: instead of loading an entire computing environment in random-access memory (RAM) each time they are executed, our pipelines only require enough memory to load the largest library container. We benefited then from a three-to-four times reduction of RAM usage when processing the MRI scans using our High-Performance Computing (HPC) cluster. We hope that our containerization approach will help other research teams reduce the computational burden of their image processing pipelines.

### 4.3 Brain segmentation

The deep network implemented in this work to segment the brain in pairs of registered structural post- mortem scans immediately achieved sufficient performance: a Dice overlap with the manual segmentation above 97 %, a true positive rate indicating that more than 97 % of the brains was correctly included in the masks, and that more than 99 % of the background was correctly discarded. As a result, no further experi- ments were conducted. We believe that this success originates from the design of the approach, focusing on small 2D patches that allowed using compact, lightweight networks with few training parameters that could be trained using dozens of thousands of patches. As a result, the networks could be thoroughly trained without over-fitting. More importantly, we think that combining T1-weighted and T2-weighted MRI signals was critical for distinguishing the brain tissues from the surrounding formalin. The deep network was also perfectly fit to distinguish the texture of the brain tissues, that were re-scaled to the same voxel resolution, from the formalin, where only low-amplitude MRI artifacts of large spatial resolution are visible. During our experiments, the possibility to build the deep networks using the CPU of a standard office computer was also extremely convenient. In the future, we will build upon our current work to reach even better performance by combining additional MRI sequences, such as other T2-weighted or Diffusion-weighted scans, and by training a single 2D patch segmentation model for the three orientations to simplify the implementation. We are convinced that similar tools could be successfully deployed in other postmortem studies. Overall, our results establish that recent Deep Network architectures can now almost perfectly segment brain tissues and distinguish brain samples from background formalin signals when combining signals extracted from com- plementary neuroimaging modalities, such as T1-weighted and T2-weighted MRIs, and many postmortem repositories offer this possibility.

### 4.4 White matter hyperintensities

The main limitation of our segmentation approach was the use of T2-weighted images to segment brain tissues in the postmortem scans. A comparison between our final brain parcellations and manually segmented white matter hyperintensity masks for brain samples where these masks could be obtained established that, on average, the volume of a grey matter region could be made by up to 17.5 % of white matter hyperintensities. The precentral cortical region and deep grey matter regions such as the caudate were the most impacted by the presence of hyperintensities. During our experiments, we mitigated this issue by swapping these volumes of white matter hyperintensity wrongly assigned to a grey matter regions between this grey matter region and the closest white matter sub-region defined by a Voronoi tessellation. This swap allowed us to produce a detailed map of the proportion of white matter hyperintensities. With the notable exception of the precentral subcortical region, all the white matter regions with noticeable proportions of hyperintensity surround the lateral ventricle, and correspond to brain regions often reported in the literature (Habes et al., 2016). Unfortunately, white matter intensity masks were only available for a subset of scans and were obtained manually. Our approach may have then accidentally introduced biases in the brain regions where hyperintensities closely resembling gray matter in T2-weighted images are frequently observed. In the future, we are planning to tackle this issue by training a Deep Network to automatically delineate the white matter hyperintensities and using the white matter hyperintensity masks generated by the network to fix the brain tissue maps before the constrained majority voting.

### 4.5 Morphometry

The women, significantly shorter than the men in our sample, present smaller brains. An atrophy with age was also observed in all the brain regions, but that effect did not reach statistical significance in the white matter cerebrum and when considering the entire brain volume. Interestingly, the volumes measured for the cerebrum white matter were similar for in vivo and postmortem scans. On the opposite, the volume of the cerebellum was under-estimated in the clinical scans. We think that this discrepancy could be explained by the MRI signal intensity drop observed in clinical MRI scans, that did not happen in the postmortem samples (Romero et al., 2017); and the fact that the cerebellum was floating far from the brain during the postmortem scans, without pressure from surrounding tissues and skull. The comparisons between in vivo and postmortem volumes in the other brain structures produced contrasted conclusions: the hippocampus was smaller in the postmortem scans, but in general, the deep grey matter appeared larger in the postmortem samples. Overall, we observed a shrinkage of the total brain volume in postmortem scans despite the larger cerebellum volumes. This could probably be explained by the shrinkage of grey matter tissues during fixation reported in the literature (Weisbecker, 2012, Birkl et al., 2016). In general, we observed stronger and more significant atrophies in the brain associated with Alzheimer’s Disease than with vascular dementia (Table 5), which is in line with the literature, such as our prior work comparing the effects of vascular dementia and Alzheimer’s Disease in MRI brain scans in vivo (Wang et al., 2024). We observed a stronger association between dementia and brain atrophy in deep grey matter regions than in the rest of the brain (Braak and Braak, 1991, Mirra et al., 1991, Knopman et al., 2003, Jellinger and Attems, 2010) (Table 5).

### 4.6 Neuropathology scores

Because of the inclusion of many brain donors in their early sixties or seventies suffering from large neuropathological burdens, the relations between neuropathology scores and brain donors’ demographics and regional volumes were challenging to model. However, our results indicate that age can be predicted as a function of neuropathology and that the predictions can be improved by including the volume of the deep grey matter regions in the model. Overall, our results are coherent with an age-related atrophy of the deep grey matter and with an accumulation of pathological proteins in the brain over time, two phenomena largely reported in the literature (Thal et al., 2002, Braak and Braak, 1991, Mirra et al., 1991). We would explain the quadratic relation between neuropathology scores and age at death observed in our sample as follows: when the neuropathology burden was moderate (sum of scores smaller than 9), neurodegeneration was not sufficient to noticeably shorten the life of the brain donors, and we observed a simple increase of neuropathology burden with age. For a large neuropathology burden on the opposite (sum of scores larger than 10), the neurodegeneration was sufficient to shorten the life of the brain donors, and our models indicate that the most extreme scores produced the most premature deaths. This interesting hypothesis will need to be further investigated in larger data sets.

### 4.7 Limits and future work

The restricted sample size is arguably the most stringent limit of the present work. Postmortem MRI scans are difficult to obtain, and we could only collect 95 pairs of structural MRI scans after visual quality assessment (Bell et al., 2008, Freund et al., 2018, Shepherd et al., 2019, Li et al., 2023). However, as the collection of brains samples keeps going on at our institution, we should gradually reach better sample sizes in the following years.

The limited quality of the clinical MRI scans available in the medical records of our brain donors also raised significant challenges. The poor spatial resolution of these scans has likely impacted the volume estimations established for the finest brain regions and, in particular, small or elongated deep grey matter regions, such as the hippocampus. This issue might explain the inconsistent differences between in and vivo and postmortem volumes observed when focusing on the hippocampus instead of the entire deep grey matter. By contrast, the morphometry analyses conducted for the entire white matter, cerebellum, and total brain volumes have been less impacted by these biases, and they present more consistent trends.

This second issue has sparked discussions about potential study protocol updates: focusing the brain donor recruitment on the cohorts of ongoing clinical studies acquiring high-quality MRI scans could guarantee the availability of in vivo and postmortem data of matching quality. But few clinical studies are appropriate for that collect since most investigations are now exploring the treatment of early symptoms with the hope of stopping neurodegenerative diseases before they progress (van Dyck et al., 2023, Swanson et al., 2021). We will need to seek new collaborators before updating our protocol.

The limited sample size offered us the possibility of reorienting and assessing the visual quality of the scans and manually improving the brain masks, but these manual steps would limit the scalability of the pipeline to larger datasets. To scale our approach, we would first alleviate the initial manual image reorientation by working with the biologists preparing the samples and the MRI technologists acquiring the scans to ensure that brain samples are always positioned with the same orientation inside the MRI scanner. We are currently exploring the use of rigid plastic containers to achieve this goal. We have demonstrated in this work that the other manual task, the editing of the brain masks, was considerably accelerated by our Deep Learning segmentation tool. A small improvement of this tool would offer the possibility of focusing the manual editing on very few difficult cases, if not completely automatizing the brain segmentation. We hope that working with more scans in the future will let us reach that milestone.

We could only incorporate white matter hyperintensity masks for half of the brain donors because the scans were very time-consuming to segment manually. We will attempt to fix this lack of white matter hyperintensity masks by dedicating more efforts to manual segmentation. In addition, the success of our patch-based approach for the segmentation of brain tissues suggests the adoption of a similar multi-modality approach to accelerate the manual delineation of the white matter hyperintensities. We will base our first experiments in that direction on our prior works addressing white matter hyperintensity segmentation in clinical structural MRI scans (Rashid et al., 2021).

In addition, the pipeline will be expanded to handle additional MRI protocols, with the aim of processing new postmortem datasets. Postmortem neuroimaging has not been standardized yet. Due to the lack of a reference approach, brain banks have developed slightly different methods to preserve, image, and dissect brain tissues (Li et al., 2023, Jonkman et al., 2019, Freund et al., 2018, Grinberg et al., 2007). This strong variability between datasets was our main incentive to develop new neuroimaging pipelines that were better adapted to our data. This design, intended to achieve the best results in our specific combination of MRI sequences, will require an adaptation to handle other types of brain samples. We believe, nonetheless, that the key components in our pipelines, such as the lightweight Deep Learning tool we developed to remove the formalin signal in our images, will be easy to adopt in new settings and will prove useful in a great variety of postmortem settings.

## 5 Conclusion

In this work, we have conducted the very first morphometry study of the neuroimaging data provided by a newly established brain bank: the South Texas Alzheimer’s Disease Research Center repository. The unique set of neuroimaging acquisition protocols implemented at our center allowed the derivation of high- quality brain masks and parcellations by combining structural MRI scans acquired using different protocols. In addition, manually delineated masks indicating white matter hyperintensities and produced by our neu- rologists were used to localize and quantify these tissue alterations to better describe their occurrences and further improve our automatic brain parcellations. Lastly, a set of clinical scans acquired while the brain donors were alive and provided by their families were parcellated and compared with the postmortem scans to derive atrophy models capturing brain fixation effects at the same time as age-related atrophy, sex differ- ences, and neurological tissues alterations. Our investigations led to a series of methodological developments that could potentially benefit numerous other postmortem studies in the future, such as the use of small and efficient Deep Network architectures to generate high-quality brain masks, tips and tricks to adapt in vivo atlases to segment postmortem samples, and the combination of registration and Deep Learning super-resolution tools to fix low-quality anisotropic scans.

Our results suggest that postmortem MRI is well-suited to study the cerebellum, which appears larger and can be more easily parcellated and studied. Overall, brain fixation tends to shrink tissues, but the cerebrum white matter appeared to be relatively preserved, while contrasting effects were observed in deep grey matter regions. We observed a quadratic relation between the neuropathology scores and the brain donors’ age at death suggesting the existence of a level of neuropathological burden where neurodegeneration becomes the leading cause of death. This hypothesis will need to be revisited in a couple of years when our ongoing brain collection will have doubled or tripled the size of the brain bank repository. In the meantime, we hope that this work will inspire other teams using postmortem MRI data and will contribute to bridging the gap between in vivo and postmortem MRI measures to improve the translation between postmortem neuropathology findings and in vivo biomarkers.

*Data and code availability statement*

The twenty brains of the OASIS-TRT-20 data set (Marcus et al., 2007) annotated according to the Desikan-Killiany- Tourville protocol (Klein and Tourville, 2012) were provided by the mindboggle website: https://mindboggle.info/data (Klein and Tourville, 2012). The Conte69 atlas (Van Essen et al., 1991) was provided by the Human Connectome Project, WU-Minn Consortium (Principal Investigators: David Van Essen and Kamil Ugurbil; 1U54MH091657) funded by the 16 NIH Institutes and Centers that support the NIH Blueprint for Neuroscience Research; and by the McDonnell Center for Systems Neuroscience at Washington University. An implementation of the postmortem processing pipeline is available on our Github page https://github.com/UTHSCSA-NAL/postmortem_morphometry_2024.

## CRediT authorship contribution statement

**Nicolas Honnorat:** Writing – original draft, Software, Methodology, Investigation, Formal analysis, Data curation, Conceptualization. **Mariam Mojtabai:** Data curation. **Karl Li:** Data curation. **Jinqi Li:** Data curation. **David Michael Martinez:** Project administration. **Tanweer Rashid:** Resources, Data curation. **Morgan Smith:** Data curation. **Margaret E Flanagan:** Funding acquisition. **Elyas Fadaee:** Data curation. **Morgan Fox Torres:** Data curation. **Mallory Keating:** Data curation. **Kevin Bieniek:** Supervision, Funding acquisition, Conceptualization. **Sudha Seshadri:** Funding acquisition. **Mohamad Habes:** Supervision, Methodology, Funding acquisition, Formal analysis, Conceptualization.

## Declaration of Competing Interest

The authors declare that they have no known competing financial interests or personal relationships that could have appeared to influence the work reported in this paper.

## Data Availability

Data will be made available on request.
